# Effect of final endodontic irrigation protocols on the microtensile bond strength of CAD/CAM endocrown materials to pulp chamber dentin: an in vitro study

**DOI:** 10.1186/s12903-026-09462-5

**Published:** 2026-08-04

**Authors:** Sary Borzangy, Sultan Ainoosah, Mohammed H. AbdElaziz, Samah Mahmoud, Redhwan Saleh Al-Gabri, Ahmed Yaseen Alqutaibi

**Affiliations:** 1 Substitutive Dental Science Department, College of Dentistry, Taibah University, Madinah, Saudi Arabia; 2 Department of Prosthodontics, Faculty of Dentistry, Ibb University, Ibb, Yemen; 3 Department of Prosthodontics, Faculty of Dentistry, National University, Ibb, Yemen

**Keywords:** Endocrown, CAD/CAM materials, Irrigation protocols, Microtensile bond strength, Pulp chamber dentin, Lithium disilicate, QMix, Sodium hypochlorite, EDTA, Antioxidant

## Abstract

**Background:**

The bond strength of computer-aided design/computer-aided manufacturing (CAD/CAM) endocrowns depends on the condition of pulp chamber dentin after endodontic treatment. Irrigation protocols can alter dentin chemistry and morphology, influencing microtensile bond strength (µTBS). This study evaluated the effect of four irrigation protocols on µTBS and failure modes of two CAD/CAM materials—lithium disilicate (LDS) and polymer-infiltrated ceramic (Vita Enamic, VE).

**Materials and methods:**

Forty extracted human mandibular first molars were randomly allocated into four irrigation protocol groups (*n* = 10/group): Group 1 (Control): distilled water; Group 2: 2.5% sodium hypochlorite (NaOCl) followed by 17% EDTA; Group 3: NaOCl + EDTA + 10% sodium ascorbate (antioxidant); and Group 4: QMix 2-in-1. After standardized endodontic treatment, teeth were restored with LDS or VE (*n* = 5 per material/group). Specimens underwent 24-hour storage and thermocycling (10,000 cycles), then were sectioned into ~ 1 mm² sticks (15 sticks per subgroup) for µTBS testing. Failure modes were analyzed using stereomicroscopy and scanning electron microscopy (SEM) . Data were analyzed using two-way ANOVA and Tukey’s HSD (α = 0.05).

**Results:**

Irrigation protocol exerted a highly significant main effect on µTBS (F = 127.01, df = 3/112, *p* < 0.001). Mean µTBS was lowest for the control group (8.6–8.9 MPa), intermediate and statistically comparable for NaOCl + EDTA (20.6–23.2 MPa) and NaOCl + EDTA + Antioxidant (23.9–25.1 MPa; *p* = 0.087), and highest for QMix (27.8–29.6 MPa; *p* < 0.001 vs. all other groups). CAD/CAM material type did not significantly affect µTBS (*p* = 0.056), and no significant protocol × material interaction was observed (*p* = 0.759). Failure mode distribution differed significantly across irrigation groups (χ² = 19.43, df = 6, *p* = 0.003).

**Conclusions:**

Within the limitations of this in vitro study, QMix was associated with the highest microtensile bond strength values for both CAD/CAM materials. The addition of sodium ascorbate did not significantly improve bond strength compared with NaOCl + EDTA under the tested conditions. CAD/CAM material type was not a statistically significant determinant of bond strength. These findings suggest that the choice of final endodontic irrigation protocol may influence adhesive bonding to pulp chamber dentin, and should be confirmed by future studies using the tooth as the statistical unit of analysis.

## Background

Endodontically treated posterior teeth frequently present with significant loss of coronal tooth structure, altered biomechanical behavior, and increased fracture susceptibility, collectively rendering their restoration one of the most demanding challenges in contemporary restorative dentistry [1–3]. Among the available restorative approaches, endocrowns fabricated from computer-aided design/computer-aided manufacturing (CAD/CAM) materials have gained considerable traction due to their capacity to utilize the pulp chamber for macromechanical retention, rely on adhesive luting for micromechanical interlocking, and in many systems enable single-visit completion [4]. The long-term clinical success of these restorations depends substantially on the quality and durability of the adhesive interface established between pulp chamber dentin and the endocrown material [5, 6]. 

Pulp chamber dentin presents a biologically and structurally distinct substrate compared with coronal or radicular dentin. The region is characterized by higher tubule density, variable peritubular dentin thickness, and the frequent presence of sclerotic or tertiary dentin, all of which can influence adhesive penetration and hybrid layer formation [7–9]. Moreover, root canal treatment necessitates the sequential application of irrigating solutions designed to achieve chemomechanical debridement, smear layer removal, and antimicrobial disinfection. Among the most employed irrigants are sodium hypochlorite (NaOCl) and ethylenediaminetetraacetic acid (EDTA), whose synergistic use constitutes the clinical standard of care [10, 11]. 

While endodontic irrigants are indispensable for clinical success, they simultaneously alter the chemical composition and surface morphology of pulp chamber dentin. NaOCl degrades the organic collagen matrix and generates reactive oxidative species that impair resin monomer infiltration and free-radical polymerization at the adhesive interface, ultimately reducing bond strength [11, 12]. EDTA, through preferential chelation of calcium ions, removes the smear layer and exposes the collagen network; [13, 14] however, antioxidants such as sodium ascorbate have been investigated as neutralizing agents capable of reversing NaOCl-induced oxidative damage [12, 15]. QMix 2-in-1 (Dentsply Sirona) integrates EDTA, chlorhexidine gluconate, and a surfactant in a single formulation, enabling simultaneous smear layer removal, antimicrobial action, and enhanced wettability [16]. 

Endodontic irrigants also modify the structural, chemical, and mechanical properties of dentin beyond the pulp chamber. Scoping-review evidence indicates that NaOCl and EDTA alter the organic and inorganic composition of coronal dentin in a concentration- and time-dependent manner, with consequences for its mechanical behavior [17]. NaOCl-induced collagen degradation and generation of reactive oxidative species can further compromise dentin bonding by interfering with resin monomer infiltration and polymerization, a mechanism increasingly recognized as central to irrigation-related bond strength changes [18]. Consistent with this, recent bond strength studies on coronal dentin report that final irrigation protocol and the sequence of chelation versus disinfection steps meaningfully affect long-term adhesive performance [19, 20]. 

Despite the extensive literature on bond strength to coronal dentin and CAD/CAM surface treatments, the specific interaction between endodontic irrigation protocols and the adhesive performance of CAD/CAM endocrown materials at the pulp chamber level, as distinct from coronal or root dentin, remains insufficiently characterized. Unlike previous investigations that examined irrigation effects on coronal or root canal dentin bonding in isolation [19–25], the present study specifically evaluated pulp chamber dentin bonded to two CAD/CAM endocrown materials under four final irrigation protocols, thereby addressing a substrate- and restoration-specific gap in the existing evidence. The present study aimed to evaluate the effect of four final irrigation protocols on the microtensile bond strength (µTBS) of lithium disilicate and polymer-infiltrated ceramic network endocrowns to pulp chamber dentin using a self-adhesive resin cement, following thermocycling aging. The null hypotheses were: (1) irrigation protocol has no significant effect on µTBS of CAD/CAM endocrowns to pulp chamber dentin; and (2) CAD/CAM material type has no significant effect on µTBS values.

## Methods

### Ethics approval and consent to participate

This study was approved by the Research Ethics Committee, College of Dentistry, Taibah University, Madinah, Saudi Arabia (Protocol No. TUCDREC/050125). All procedures involving extracted human teeth were conducted in accordance with relevant institutional guidelines and regulations and followed the principles of the Declaration of Helsinki for research involving human-derived materials. Teeth were collected following routine clinical extractions and were anonymized prior to use. The requirement for informed consent was waived by the Research Ethics Committee, College of Dentistry, Taibah University, Madinah, Saudi Arabia, in accordance with institutional regulations governing the use of de-identified extracted human biological materials for research purposes. The study was conducted in accordance with the modified CONSORT for reporting pre-clinical in vitro studies on dental material as proposed by Faggion [26]. 

Forty extracted human mandibular first molars, extracted for reasons unrelated to this study (e.g., orthodontic or periodontal indications) from adult patients aged 18–60 years, were selected based on standard inclusion criteria: complete root formation, intact caries-free crowns without prior endodontic treatment. All teeth were used within 3 months of extraction. Teeth were stored in 0.1% thymol solution at 4 °C and examined stereomicroscopically at 20–40× prior to use.

### Sample size calculation

Sample size was calculated a priori using G*Power software (version 3.1.9.7) for a two-way fixed-effects ANOVA, with irrigation protocol (4 levels) and CAD/CAM material type (2 levels) as independent variables and µTBS as the primary outcome. Based on previous µTBS data [21], a conservative effect size of f = 0.40 was adopted. At α = 0.05 and 80% power, a minimum of 10 sticks per subgroup was required (total *N* = 80). This was increased to 15 sticks per subgroup to compensate for potential specimen loss during sectioning, yielding 5 teeth per material per irrigation group. Because multiple sticks were obtained from each tooth, this calculation did not account for within-tooth clustering, and the study was powered at the stick level rather than the tooth level. The implications of this analytical approach are acknowledged in the Discussion and Limitations sections.

### Study design and randomization

The study followed a two-factor in vitro design (irrigation protocol: 4 levels; CAD/CAM material: 2 levels). Forty teeth were randomly allocated to four irrigation groups (*n* = 10/group), further subdivided by material (*n* = 5 teeth/subgroup; 15 sticks/subgroup after sectioning). Failure mode assessment was performed by two independent blinded evaluators (Cohen’s kappa = 0.87, indicating almost perfect agreement).

### Standardized tooth preparation and endodontic procedures

All teeth were decoronated perpendicular to the long axis at the cementoenamel junction. Endocrown preparations were executed with a tapered carbide fissure bur (Komet H33L) providing a uniform 3 mm axial wall height with a 360° supragingival butt-joint finish line (6–8° divergence). Canals were instrumented with HyFlex CM rotary files (Coltene Whaledent), with mesial canals prepared to #35/0.04 and distal to #40/0.04 taper. During mechanical instrumentation, canals were irrigated with 5 mL 2.5% NaOCl after each file change, followed by a final flush of 5 mL 17% EDTA and 5 mL distilled water prior to obturation; this standardized intra-instrumentation irrigation protocol was identical across all experimental groups and preceded the final irrigation protocols described below. Canals were then obturated with gutta-percha (Dentsply Sirona) and AH Plus Bioceramic Sealer (Dentsply Sirona, Konstanz, Germany) via lateral condensation.

### Irrigation protocols

Upon completion of canal instrumentation, specimens received one of four final irrigation regimens:

- Group 1 (Control): 5 mL distilled water for 60 s.
- Group 2 (NaOCl + EDTA): 5 mL 2.5% NaOCl for 60 s, then 5 mL 17% EDTA for 60 s, followed by 5 mL distilled water flush.
- Group 3 (NaOCl + EDTA + Antioxidant): Sequence as Group 2, then 5 mL 10% sodium ascorbate for 120 s, then 5 mL distilled water flush.
- Group 4 (QMix): 5 mL QMix 2-in-1 (Dentsply Sirona) for 120 s, then 5 mL distilled water flush.

All irrigants were delivered via a 30-gauge side-vented needle positioned 1–2 mm short of working length.

### CAD/CAM endocrown fabrication and cementation

**Fig. 1:**
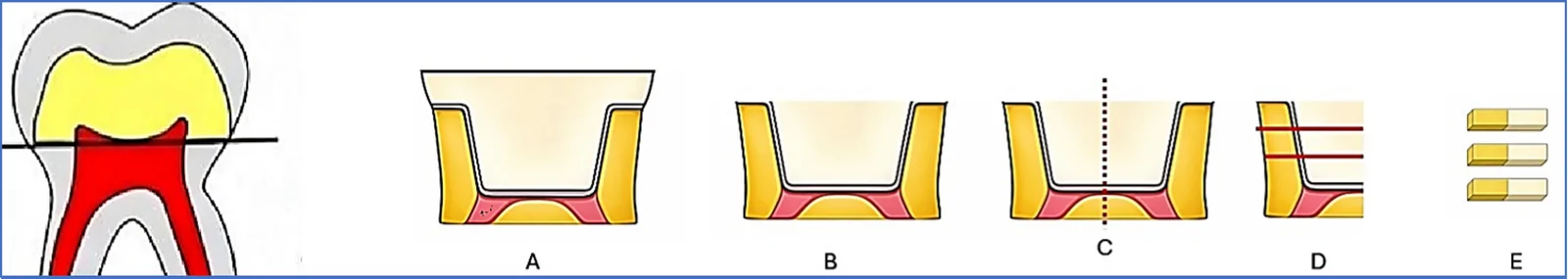
Schematic representation of the standardized tooth preparation and sectioning protocol for the microtensile bond strength (µTBS) test: (**A**, **B**) standardized tooth preparation design and endocrown restoration; (**C**, **D**) sequential specimen preparation (vertical and horizontal cuts); (**E**) endocrown-dentin beams (1 ± 0.05 mm²) for µTBS testing

Prepared teeth were digitally scanned (TRIOS 3, 3Shape). Endocrowns were designed with 60 μm cement spacing and 2 mm uniform occlusal thickness (Fig. 1A and B). LDS restorations (IPS e.max CAD) underwent crystallization/glazing; VE restorations (Vita Enamic, Vita Zhanfabrik) were milled from prefabricated blocks using a 5-axis machine (ED5X; EMAR). Intaglio surfaces of LDS were etched with 5% hydrofluoric acid (HF) (IPS Ceramic Etching Gel; Ivoclar Vivadent) for 20 s; VE surfaces for 60 s, followed by silane application. Dentin surfaces were gently air-dried to a visibly moist (non-desiccated) condition prior to cementation, avoiding both over-drying and pooling of moisture. RelyX Unicem 2 (3 M ESPE, Seefeld, Germany) was applied and light-cured for 20 s per surface (1200 mW/cm² LED curing light) following a 2-second tack-cure to facilitate excess removal, then cemented under constant 5 kg load for 5 min using a custom device to achieve uniform film thickness.

### Aging and microtensile testing

Specimens were stored in distilled water at 37 °C for 24 h, then thermocycled for 10,000 cycles (5–55 °C; ISO 11405:2015). Assemblies were serially sectioned perpendicular to the occlusal plane with a low-speed diamond saw under water cooling. Mesiodistal and buccolingual cuts were made (Fig. 1C and D) to produce bonded sticks (~ 1 × 1 mm²) (Fig. 1E). Sticks were tested on a universal testing machine (Lloyd Instruments Ltd) at 0.5 mm/min crosshead speed (ISO 29022:2013). Any specimen that debonded during sectioning or handling before formal testing was defined as a pre-test failure and recorded by subgroup.

### Failure mode analysis

Fractured specimens were examined at 40× magnification using a stereomicroscope (AmScope 3.5; Irvine, CA, USA) by two independent blinded evaluators. Failure modes were classified as adhesive, when failure occurred at the dentin–cement or cement–restoration interface; cohesive, when failure occurred within dentin, resin cement, or restorative material; or mixed, when both adhesive and cohesive features were present. The presence and number of pre-test failures were also recorded for each subgroup. Representative fractured specimens from each irrigation/material subgroup were selected for SEM analysis, dehydrated in an ascending ethanol series (50–100%), air-dried, mounted on aluminum stubs, and sputter-coated with gold-palladium prior to imaging. Specimens were examined using scanning electron microscopy (SEM) at 20 kV to assess interfacial morphology and support interpretation of the bond strength findings.

### Statistical Analysis

Statistical analyses were performed using IBM SPSS Statistics (Version 26.0) and verified with Python (SciPy/Statsmodels). Normality was assessed with the Shapiro–Wilk test. Two-way ANOVA evaluated main effects of irrigation protocol and material type, and their interaction. Pairwise comparisons used Tukey’s HSD. Effect sizes were reported as partial η². Failure mode frequencies were compared with chi-square test. α = 0.05.

## Results

### Microtensile bond strength

**Fig. 2:**
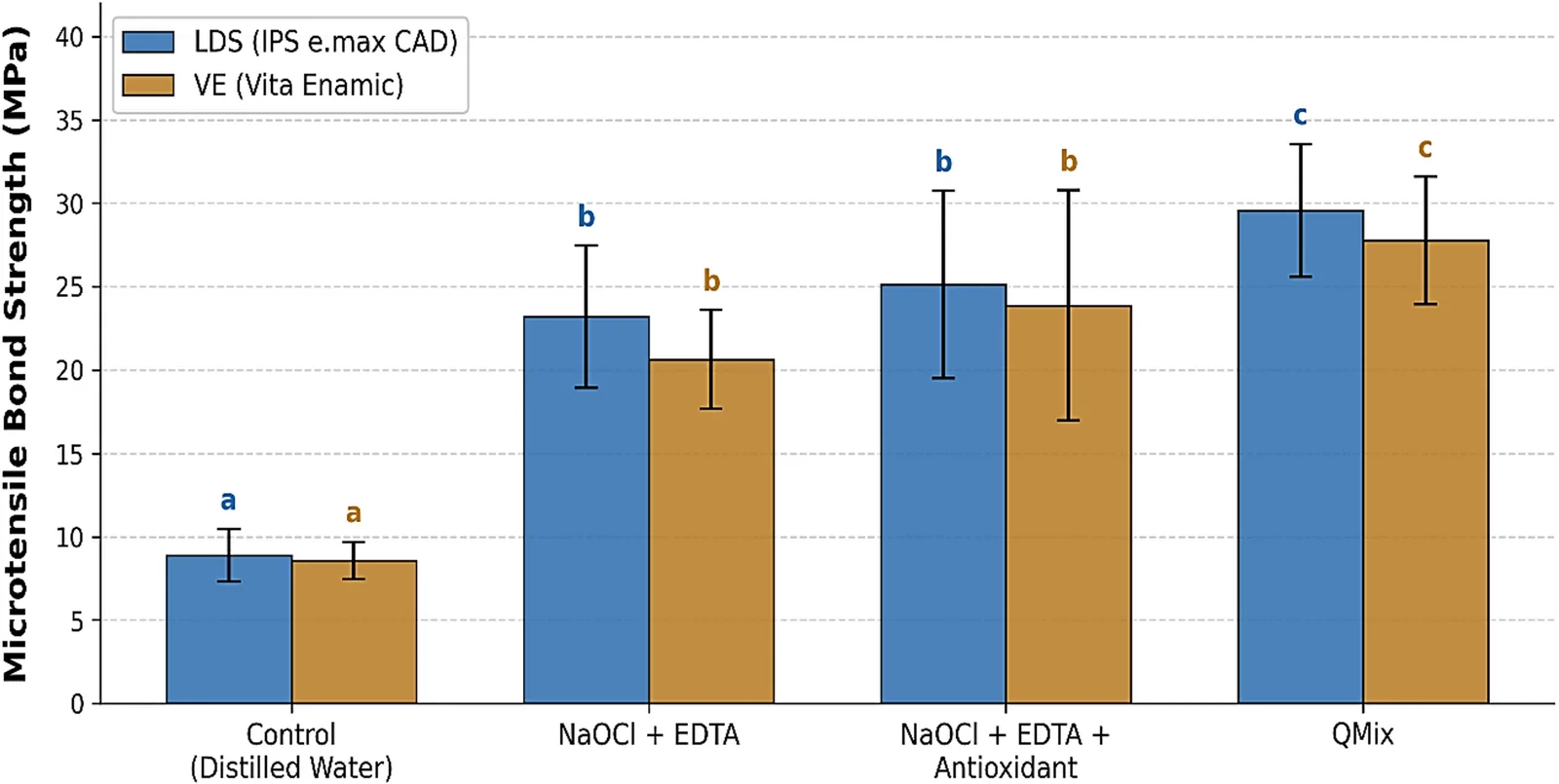
Mean microtensile bond strength (µTBS ± SD) for lithium disilicate (LDS) and Vita Enamic (VE) endocrown materials following each irrigation protocol. Different lowercase letters indicate statistically significant differences between irrigation groups (Tukey’s HSD, *p* < 0.05). Groups sharing the same letter do not differ significantly

**Fig. 3:**
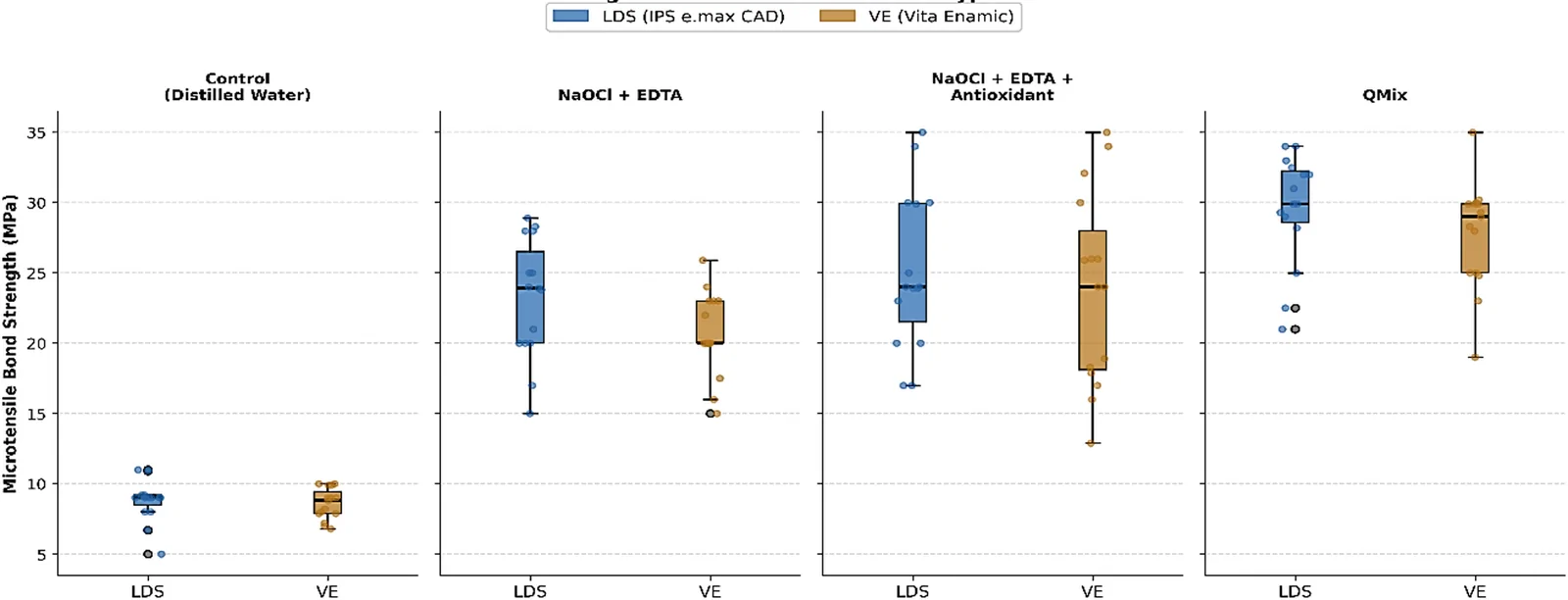
Box plots showing individual data distribution for each irrigation protocol and material combination. Data points represent individual stick values (*n* = 15/subgroup). Horizontal lines indicate medians; boxes represent interquartile ranges

Descriptive statistics and Tukey homogeneous subsets are presented in Table 1. The individual data distribution is illustrated in Figs. 2 and 3. Two-way ANOVA results are presented in Table 2.

**Table 1:** Microtensile bond strength (µTBS) of lithium disilicate (LDS) and Vita Enamic (VE) endocrown materials bonded to pulp chamber dentin after different final irrigation protocols. Values are presented as mean ± standard deviation (SD) in MPa (*n* = 15 sticks per subgroup, derived from five teeth)

Irrigation Protocol	Material	µTBS, mean ± SD (MPa)	*n*
Control (Distilled Water)	LDS	8.87 ± 1.58^a^	15
	VE	8.57 ± 1.10^a^	15
NaOCl + EDTA	LDS	23.19 ± 4.26^b^	15
	VE	20.63 ± 2.97^b^	15
NaOCl + EDTA + Antioxidant	LDS	25.11 ± 5.61^b^	15
	VE	23.87 ± 6.90^b^	15
QMix 2-in-1	LDS	29.55 ± 3.97^c^	15
	VE	27.75 ± 3.83^c^	15

Different lowercase superscript letters (a–c) indicate statistically significant differences among irrigation protocols in the overall comparison pooled across both CAD/CAM materials (Tukey’s HSD, p < 0.05). Mean values sharing the same superscript letter are not significantly different. Superscripts do not indicate comparisons between LDS and VE within the same irrigation protocol

*LDS *lithium disilicate (IPS e.max CAD), *VE *Vita Enamic (polymer-infiltrated ceramic network), *µTBS *microtensile bond strength, *SD *standard deviation

**Table 2:** Two-way analysis of variance (ANOVA) results for microtensile bond strength (µTBS) as a function of irrigation protocol and CAD/CAM material type

Source of Variation	df	SS	F	*p*-value	Partial η²
Irrigation Protocol	3	6671.02	127.01	**< **0.001	0.773
CAD/CAM Material	1	65.42	3.74	0.056	0.032
Protocol × Material (Interaction)	3	20.59	0.392	0.759	0.010
Residual (Error)	112	1960.89	—	—	—

The interaction term (Protocol × Material) was not statistically significant, indicating a consistent pattern across both materials

*df *degrees of freedom, *SS *sum of squares, *F *F-statistic, *partial η² *partial eta-squared (effect size)

Irrigation protocol exerted a highly significant main effect on µTBS (F = 127.01, df = 3/112, *p* < 0.001, partial η² = 0.773), indicating that irrigation protocol explains 77.3% of the variance in bond strength—a very large effect size. CAD/CAM material type did not reach statistical significance as a main effect (F = 3.74, df = 1/112, *p* = 0.056, partial η² = 0.032). The protocol × material interaction was also non-significant (F = 0.392, df = 3/112, *p* = 0.759), indicating that the relative ranking of irrigation protocols was consistent across both materials.

Tukey’s HSD identified three homogeneous subsets. The control group yielded the lowest µTBS values (LDS: 8.87 ± 1.58 MPa; VE: 8.57 ± 1.10 MPa), significantly lower than all other groups (*p* < 0.001). The NaOCl + EDTA (LDS: 23.19 ± 4.26 MPa; VE: 20.63 ± 2.97 MPa) and NaOCl + EDTA + Antioxidant (LDS: 25.11 ± 5.61 MPa; VE: 23.87 ± 6.90 MPa) groups were statistically comparable to each other (*p* = 0.087), forming a second homogeneous subset that was significantly lower than QMix (*p* < 0.001) and significantly higher than control (*p* < 0.001). The QMix group exhibited the highest µTBS for both materials (LDS: 29.55 ± 3.97 MPa; VE: 27.75 ± 3.83 MPa), significantly exceeding all other groups (*p* < 0.001 vs. Control; *p* < 0.001 vs. NaOCl + EDTA and NaOCl + EDTA + Antioxidant). Within each irrigation group, LDS and VE bond strengths did not differ significantly (all *p* > 0.05 by independent t-test).

### Failure mode analysis

**Fig. 4:**
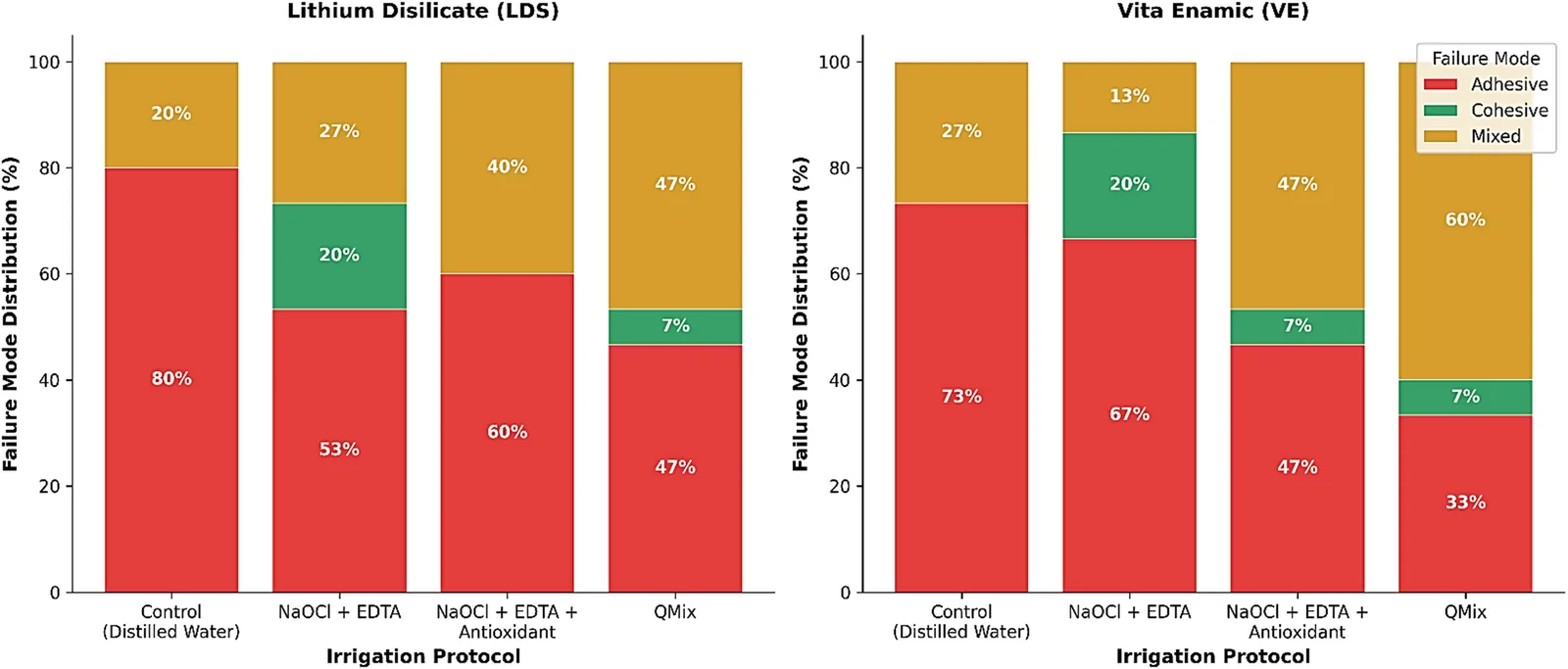
Stacked bar charts showing the percentage distribution of failure modes (adhesive, cohesive, mixed) for lithium disilicate (LDS) and Vita Enamic (VE) in each irrigation protocol group

The distribution of failure modes is presented in Fig. 4. No pre-test failures occurred in any experimental subgroup: 0/15 specimens in each of the eight subgroups and 0/120 specimens overall. All 120 sticks survived sectioning and specimen handling and were successfully tested to failure, yielding valid µTBS values and failure-mode classifications. Therefore, no zero-value imputation for pre-test failures was required. Chi-square analysis revealed a significant difference in failure-mode distribution across irrigation groups (χ² = 19.43, df = 6, *p* = 0.003).

In the control group, adhesive failures were predominant (LDS: 80.0%; VE: 73.3%), with no cohesive failures recorded for either material, consistent with weak interfacial integration. In the NaOCl + EDTA group, cohesive failures appeared (LDS: 20.0%; VE: 20.0%), with lower adhesive failure rates. The NaOCl + EDTA + Antioxidant group showed further reduction in adhesive failures and higher mixed failure proportions. The QMix group demonstrated the highest proportion of mixed failures for VE (60.0%) and substantial mixed failures for LDS (46.7%), with comparably low pure adhesive failure rates, reflecting superior interfacial integration.

### Scanning electron microscopy

**Fig. 5:**
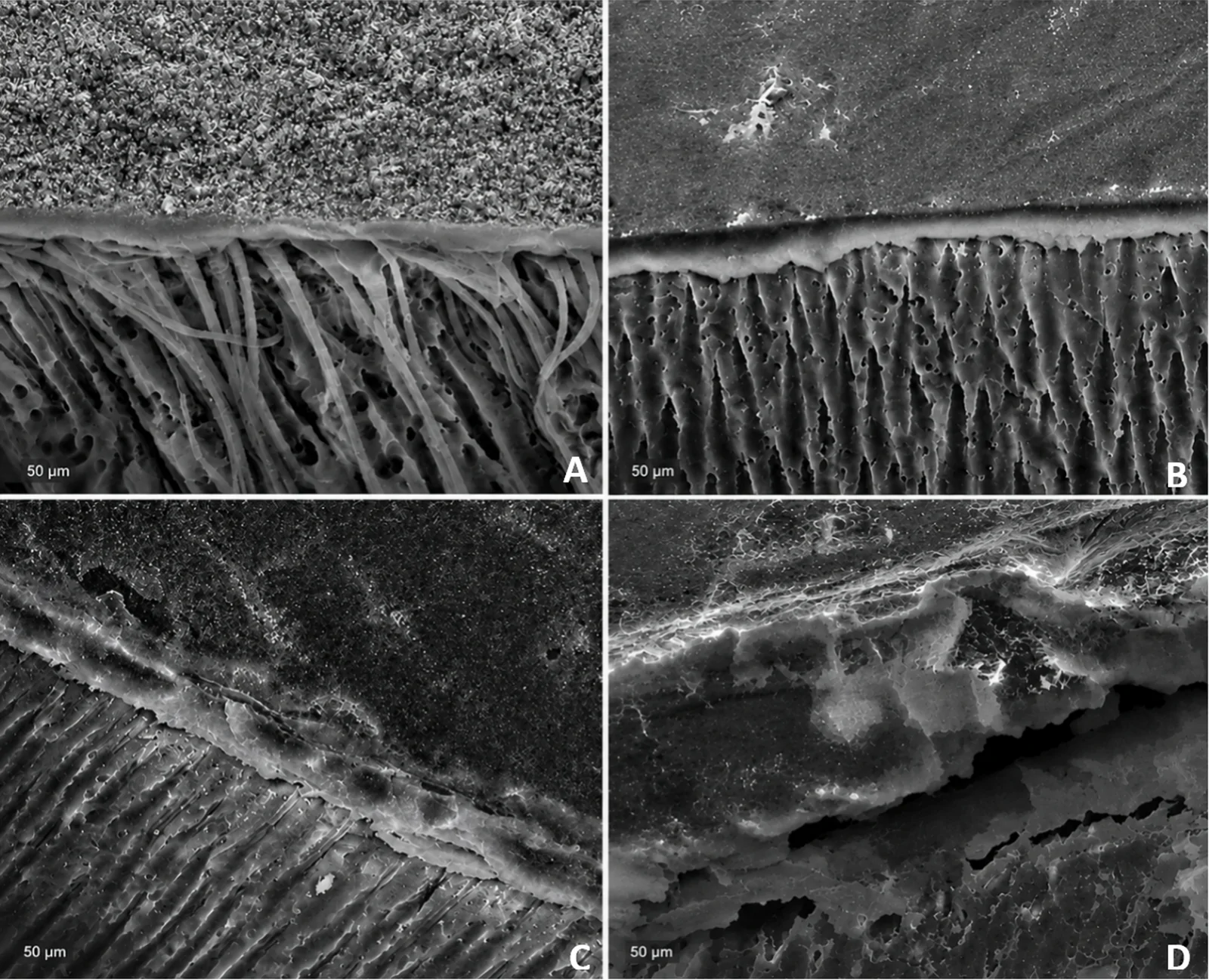
Representative SEM micrographs of the dentin–endocrown interface in the experimental groups: (**A**) QMix, (**B**) NaOCl + EDTA, (**C**) NaOCl + EDTA + antioxidant, and (**D**) distilled-water control. Original magnification: 1000×; scale bar: 50 μm in each panel

SEM evaluation of fractured interfaces (Fig. 5) revealed distinct ultrastructural differences across irrigation groups. The QMix group showed more continuous interfacial adaptation and more evident resin tag formation extending into dentinal tubules, consistent with the superior µTBS values observed for this group. Control specimens displayed an incomplete interfacial adaptation with minimal resin tag formation and evidence of residual smear layer occlusion. Qualitative differences between the NaOCl + EDTA and NaOCl + EDTA + Antioxidant groups were not consistently distinguishable on SEM examination.

## Discussion

This in vitro study evaluated the effect of four final endodontic irrigation protocols on the µTBS of two CAD/CAM endocrown materials bonded to pulp chamber dentin. Irrigation protocol exerted a statistically significant main effect on µTBS (*F* = 127.01, *p* < 0.001, partial η² = 0.773), with QMix producing the highest bond strength values, whereas the main effect of CAD/CAM material type did not reach statistical significance at the predefined α level (*p* = 0.056), and no statistically significant protocol × material interaction was detected (*p* = 0.759). Accordingly, the first null hypothesis was rejected, whereas the second null hypothesis was not rejected under the tested conditions. Nevertheless, the material-related *p*-value was close to the significance threshold and should not be interpreted as evidence that LDS and VE are equivalent; further adequately powered studies are needed to clarify whether material type influences µTBS. Because multiple sticks were obtained from each tooth without accounting for within-tooth clustering, the reported *p*-values, effect sizes, and precision estimates warrant cautious interpretation, an issue examined further under Limitations.

The superior bond strength achieved with QMix is likely attributable to its combined chelating action and surfactant-mediated reduction in surface tension, which together enhance removal of residual surface debris, improve dentin wettability, and facilitate resin cement adaptation [16, 27]. Published evidence on QMix, however, is not uniform: Elnaghy reported higher bond strength of glass-fiber posts to root dentin after QMix irrigation than after 5.25% NaOCl [22], whereas Barreto et al. reported lower bond-strength values with QMix than with several alternative irrigation protocols [28]. This apparent inconsistency suggests that the bonding effect of QMix is substrate- and protocol-dependent; the present findings therefore indicate an association between QMix and higher µTBS under the specific conditions tested, rather than a universal bonding advantage.

Consistent with this interpretation, the control group’s lowest µTBS values (approximately 8.6–8.9 MPa) were attributable to the persistence of an intact smear layer over dentinal tubule orifices. Because distilled water lacks chelating capacity, it is unlikely to remove the smear layer generated during preparation; consequently, this residual layer likely acted as a weak intermediate substrate between dentin and resin cement, physically obstructing resin monomer infiltration and reducing micromechanical interlocking [7, 29, 30]. This interpretation is further supported by the predominance of adhesive failures in the control group (73–80%), consistent with prior investigations on untreated pulp chamber dentin [31, 32]. 

Final irrigation with NaOCl + EDTA confirmed that smear layer removal substantially benefits adhesive bonding, although residual oxidative species generated by the preceding NaOCl step may persist on the dentin surface despite subsequent EDTA chelation, potentially compromising resin polymerization and limiting the bond strength gains achievable through smear layer removal alone. This finding contrasts with a substantial body of prior evidence indicating that endodontic irrigation, particularly NaOCl, can impair rather than enhance dentin bond strength [7, 11, 18, 33]. Several methodological factors may account for this discrepancy. First, all irrigants in this study were applied as a final rinse after instrumentation rather than during extended canal disinfection, limiting NaOCl contact time and oxidative deposition; the pulp chamber dentin substrate is also more superficial and less tubule-dense than root canal dentin, and may therefore be less susceptible to irrigant-induced collagen degradation [11, 12, 33]. Second, differences in irrigant composition and application sequence across studies — for example, single-step formulations combining chelating agents and surfactants versus sequential NaOCl–EDTA protocols or irrigants used throughout instrumentation — may differentially affect dentin wettability and surface cleanliness [16, 27, 33]. Third, the self-adhesive resin cement used in this study, applied after material-specific surface pretreatment, differs from the etch-and-rinse, self-etch, or multistep adhesive systems evaluated in several previous investigations, and such differences in bonding strategy may independently influence bond strength [34, 35]. Finally, methodological heterogeneity in bond-strength testing — microtensile, microshear, and push-out tests differ in specimen geometry, stress distribution, and bonded substrate — further complicates direct comparison across the literature [7, 22, 28]. Taken together, the higher µTBS values observed with NaOCl + EDTA and QMix in the present study should be interpreted as specific to the present experimental conditions rather than as a general contradiction of prior findings.

The addition of sodium ascorbate did not significantly augment bond strength in this study (*p* = 0.087 vs. NaOCl + EDTA). Although prior literature has reported antioxidant benefits [15, 36–40], the absence of a significant effect here may reflect the shorter 120-second antioxidant application used, relative to the longer contact times (10 min or more) required in some previous protocols, as well as the greater variability in the antioxidant group’s SD (5.61–6.90 MPa), which reduced statistical power.

Two CAD/CAM materials were included as an exploratory component of the study to assess whether differences in composition and material-specific surface pretreatment modified the effect of the irrigation-conditioned dentin substrate. Neither the main effect of material type nor the protocol × material interaction reached statistical significance; however, because the material-related *p*-value approached the predefined significance threshold (*p* = 0.056), these findings do not establish equivalence between LDS and VE, nor do they exclude a potentially meaningful material effect — they indicate only that no clear evidence was found that the relative influence of irrigation protocol differed between the two materials. Both materials received material-specific pretreatment before cementation, which may have minimized differences at the restoration–cement interface and contributed to the non-significant material effect; previous evidence indicates that dentin-side bonding strategies and the type of resin luting material can independently influence the bond strength of CAD/CAM restorations to dentin [34, 35]. These results suggest that dentin conditioning contributed substantially to the measured µTBS, without establishing the dentin-side interface as the sole or definitive limiting factor. Future studies with larger, tooth-level samples should evaluate additional CAD/CAM materials and adhesive or luting strategies while accounting for within-tooth clustering.

The progressive shift from predominantly adhesive failures in the control group toward mixed failures in the QMix and antioxidant groups corroborates the µTBS findings. Adhesive failures generally indicate that the bonded interface is the weakest link, whereas mixed failures suggest that stresses are distributed beyond the interface into the cement or dentin substrate [41, 42]. The high proportion of mixed failures observed with QMix (60% in VE, 46.7% in LDS) is therefore consistent with improved resin–dentin interfacial integration [7, 31, 41, 43]. 

Clinically, these findings suggest that final irrigation protocols selected before endocrown cementation should account not only for endodontic disinfection but also for their effect on subsequent adhesion. QMix may offer a simplified final-irrigation strategy capable of producing a dentin surface more favorable for adhesive luting; however, because this was an in vitro study using one resin cement and short-term aging, direct clinical recommendations should be made cautiously.

Several limitations should be acknowledged. Most importantly, the statistical analysis treated each microtensile stick as an independent experimental unit, although 15 sticks were obtained from only five teeth per subgroup. Sticks originating from the same tooth may be correlated; treating them as independent observations may therefore introduce pseudoreplication, inflating the apparent degrees of freedom and statistical power relative to a tooth-level or mixed-effects analysis. Because the final analytical dataset did not retain identifiers linking each tested stick to its parent tooth, retrospective tooth-level aggregation or hierarchical reanalysis was not feasible; consequently, the magnitude, precision, and statistical significance of the reported effects should be interpreted with caution. Future studies should preserve specimen-to-tooth linkage and analyze µTBS data using tooth-level means or linear mixed-effects models with tooth included as a random effect.

Additional limitations include the in vitro design, the relatively short sodium ascorbate application time, the assessment of only two CAD/CAM materials with a single resin cement and luting strategy, the restriction to pulp chamber dentin, and the use of laboratory aging rather than long-term clinical evaluation. Future investigations should incorporate hierarchical statistical designs, fatigue loading, extended aging, additional restorative materials and luting protocols, and ultimately clinical studies.

## Conclusions

Within the limitations of this in vitro study, the following conclusions can be drawn:

1. QMix 2-in-1 irrigation produced the highest microtensile bond strength to pulp chamber dentin for both LDS and VE endocrown materials, significantly exceeding all other protocols.
2. NaOCl + EDTA and NaOCl + EDTA + Antioxidant groups produced statistically comparable bond strengths; the antioxidant step did not yield a statistically significant additive improvement under the conditions tested.
3. These findings suggest that active dentin conditioning may improve adhesion to pulp chamber dentin under the tested conditions.
4. CAD/CAM material type (LDS vs. VE) was not a statistically significant determinant of bond strength (*p* = 0.056), and the interaction between irrigation protocol and material was non-significant.
5. Failure mode distribution shifted significantly across irrigation groups, with QMix producing the highest proportion of favorable mixed failures.

## Data Availability

Datasets are available from the corresponding author upon reasonable request.

## Abbreviations

ANOVA: Analysis of variance
CAD/CAM: Computer-aided design/computer-aided manufacturing
EDTA: Ethylenediaminetetraacetic acid
HF: Hydrofluoric acid
HSD: Honestly significant difference
LDS: Lithium disilicate glass-ceramic
MPa: Megapascal
NaOCl: Sodium hypochlorite
SD: Standard deviation
SEM: Scanning electron microscopy
VE: Vita Enamic
µTBS: Microtensile bond strength
